# In Vitro Investigation of the Antiproliferative and Antimetastatic Effects of Atorvastatin: A Focus on Cervical and Head and Neck Cancers

**DOI:** 10.3390/pharmaceutics17101253

**Published:** 2025-09-24

**Authors:** Hiba F. Muddather, Noémi Bózsity, György T. Balogh, Zsuzsanna Schelz, István Zupkó

**Affiliations:** 1 Institute of Pharmacodynamics and Biopharmacy, Faculty of Pharmacy, University of Szeged, Eötvös u. 6, 6720 Szeged, Hungary; hiba.161991@hotmail.com (H.F.M.); bozsity-farago.noemi@szte.hu (N.B.); balogh.gyorgy.tibor@semmelweis.hu (G.T.B.); 2 Department of Clinical Pharmacy and Pharmacy Practice, Faculty of Pharmacy, University of Gezira, Hospital Street, Wad Medani 21112, Sudan; 3 Department of Pharmaceutical Chemistry, Faculty of Pharmacy, Semmelweis University, Hőgyes Endre u. 9, 1092 Budapest, Hungary; 4 Department of Chemical and Environmental Process Engineering, Budapest University of Technology and Economics, Műegyetem rkp. 3, 1111 Budapest, Hungary

**Keywords:** atorvastatin, rosuvastatin, antiproliferative, antimetastatic, apoptosis, repurposing

## Abstract

**Background/Objectives**: In spite of substantial treatment progress, cancer persists as a leading health challenge. With the slow advancement in developing new anticancer agents, drug repurposing provides a promising strategy to enhance cancer therapy. This study investigates the antiproliferative and antimetastatic properties of two 3-Hydroxy-3-methylglutaryl coenzyme A (HMG-CoA) reductase inhibitors, atorvastatin and rosuvastatin, which represent lipophilic and hydrophilic statins, respectively. **Methods**: Growth inhibition was evaluated in a panel of human cancer cells using the standard MTT assay. Apoptotic effects were determined through flow cytometry, caspase-3 activity assay, mitochondrial membrane potential assessment, and Hoechst/Propidium iodide fluorescent double staining. Migration and invasion assays were conducted using wound-healing and Boyden chamber assays, respectively. **Results:** Atorvastatin demonstrated more pronounced growth-inhibitory effects than rosuvastatin, with the IC_50_ values in the range of 2.57–61.01 µM. Atorvastatin exhibited both biochemical and morphological indicators of apoptosis. Flow cytometry revealed cell cycle disruptions and increased sub-G1 apoptotic populations in HPV-positive oral squamous carcinoma cells (UPCI-SCC-154) and HPV-negative cervical cancer cells (C33A). Atorvastatin also significantly inhibited cell migration and invasion in the tested cell lines. **Conclusions**: Our results highlight the promising anticancer potential of atorvastatin in cervical cancer and oral squamous carcinoma cells. However, these findings are limited to in vitro models and warrant further in vivo validation.

## 1. Introduction

Cancer persists as a major contributor to global mortality despite the great efforts that have been made to improve cancer therapies over the years [1]. The global cancer impact in terms of morbidity and mortality continues to grow [2,3], and by 2050, the worldwide cancer burden is expected to rise by 77% from 2022 [4]. The emergence of drug resistance in tumor cells to available anticancer drugs has decreased the antitumor efficacy of therapeutic agents [5,6], underscoring the urgent need to develop new therapies or anticancer drugs. However, creating a new anticancer drug to improve outcomes is costly and could take more than a decade from discovery to approval [7,8]. One strategy to reduce this obstacle is repurposing approved pharmaceuticals that demonstrate promising antiproliferative or antimetastatic efficacy [9]. Drug repurposing has served as a strategy in cancer management for several decades. Examples of repurposed drugs in cancer management include zoledronic acid and thalidomide [10,11]. Although initially developed for other indications, these drugs have become integral to cancer therapy.

3-Hydroxy-3-methylglutaryl coenzyme A (HMG-CoA) reductase inhibitors, widely referred to as statins, competitively block the enzyme’s active site and thereby prevent the transformation of HMG-CoA into mevalonate, which is the rate-limiting step in cholesterol biosynthesis [12]. In addition, they interfere with the biosynthesis of isoprenoid intermediates [13] that facilitate the post-translational modification of small monomeric GTPases, such as Ras, Rho, and Rac [14]. As part of cardiovascular risk management, statins are utilized not only to treat dyslipidemia but also to prevent primary and secondary cardiovascular disorders [15,16]. Recently, emerging data have highlighted the in vitro anticancer properties of statins [17] on prostate [18,19,20], breast [21], liver [22,23], and colon cell lines [24,25]. Observational research demonstrated the potential role of statins in cancer suppression. A cohort study found that statin use was profoundly associated with a lower risk of breast and cervical cancer incidence and with lower mortality of breast and gynecologic cancers [26]. Moreover, the association of atorvastatin with radiotherapy, targeted treatments, and zoledronic acid provided better results in cancer patients [27]. Another study among patients with cervical cancer showed that the use of lipophilic statins was associated with superior survival rates compared to non-users [28]. Statin use was associated with better outcomes in patients with head and neck cancer and dyslipidemia compared to patients with normal lipid profiles and patients with dyslipidemia without statin use [29]. These findings support the rationale of statin use for cancer patients. Controversially, a meta-analysis of 35 randomized controlled studies failed to prove any protective effect of statins against cancer development or occurrence [30]. Furthermore, a cohort of 180,855 female participants in the United Kingdom revealed a positive correlation between statin use and cervical cancer risk. Still, no association was observed for ovarian or endometrial malignancies [31]. Additionally, clinical data demonstrated a possible association between statin use and carcinogenesis [32].

The antitumor effects of statins involve diverse molecular mechanisms, particularly the regulation of cell growth, apoptosis, cell cycle dynamics, and the metastatic capacity of cancer cells [33]. In head and neck cancers, statins, by targeting the mevalonate pathway, have demonstrated the potential to inhibit radiation resistance, indicating that the mevalonate pathway may present a potential target for overcoming treatment resistance [34]. In addition, statins decrease the capacity of cancer cells to metastasize through multiple mechanisms, including the endothelial expression of adhesion mediators, such as E-selectin, and inhibiting endothelial–mesenchymal transition (EMT) [35,36,37]. Statins can inhibit angiogenesis by attenuating the generation of vascular endothelial growth factor (VEGF), a key mediator of angiogenesis, induced by cytokines and endothelial cell proliferation [38,39]. On the other hand, statins could exhibit their antitumor effects independent of their influence on the mevalonate pathway [40]. Statins also modulate immune responses [41,42].

Statins show antineoplastic effects in several cancer types; therefore, we hypothesize that these drugs exert an impact on cervical cancer and oral squamous cell carcinomas (OSCC) as well. This study aims to fill this gap by evaluating the effects of atorvastatin and rosuvastatin, as representative and most commonly prescribed lipophilic and hydrophilic statins, on cellular proliferation, apoptotic activity, cell cycle progression, cell motility, and invasion in cervical and OSCC cells, in which the HPV infection has been widely accepted as a crucial factor in their carcinogenesis.

## 2. Materials and Methods

### 2.1. Chemicals and Cell Lines

Atorvastatin and rosuvastatin were obtained from Gedeon Richter Plc. (Budapest, Hungary). Stock solutions of the compounds used for the experiments were prepared in dimethylsulfoxide (DMSO) at 10 mM concentration and subsequently were further diluted in the appropriate culture media to obtain the desired concentrations. The final DMSO concentration was kept at or below 1% (100-fold dilutions) to ensure that solvent exposure did not influence cell proliferation. Cells were mainly procured from the European Collection of Authenticated Cell Cultures (ECACC, Salisbury, UK). SiHa and C33A cell lines, however, were acquired from the American Tissue Culture Collection (ATCC, Manassas, VA, USA), and OSCC cells were purchased from DSMZ—German Collection of Microorganisms and Cell Cultures GmbH (Braunschweig, Germany). For maintenance, cultures were grown in Eagle’s Minimal Essential Medium (EMEM), enriched with 10% heat-inactivated fetal bovine serum (FBS), 1% non-essential amino acid (NEAA) supplement, and a mixture of 1% penicillin, streptomycin, and amphotericin B. Of note, 1% L-glutamine was additionally supplemented in the medium used for UPCI-SCC-154 cell maintenance. Professor Mónika Kiricsi from the University of Szeged, Hungary, kindly gifted the non-cancerous human fibroblast cell line (MRC-5). These cells were cultured in low-glucose Dulbecco’s Modified Eagle Medium (DMEM) containing 20% FBS, 1% antibiotic-antimycotic solution, and 2% L-glutamine. Cultures were kept at 37 °C under a humidified atmosphere with 5% CO_2_. Media were purchased from Capricorn Scientific Ltd. (Ebsdorfergrund, Germany), and the supplements used in the media preparation were obtained from Lonza Group Ltd. (Basel, Switzerland). Experimental materials, including chemicals and kits, were acquired from Merck Life Science Ltd. (Budapest, Hungary) unless otherwise specified.

### 2.2. Cell Antiproliferative MTT Assay

A standard MTT assay was carried out to evaluate the antiproliferative properties of atorvastatin and rosuvastatin on a panel of human cancer cell lines [43]. Briefly, 100 µL/well of cell suspensions was seeded into 96-well plates at a density of 5000 cells per well, except for C33A and MRC-5 cells, which were seeded at 10,000 cells per well, followed by overnight incubation under typical cell culture conditions. Different concentrations of the test compounds, ranging from 0.3 to 100 µM, were applied to the cells. The control group contained cells incubated in the medium. After 72 h of incubation, 20 µL MTT reagent [5 mg/mL in phosphate-buffered saline (PBS)] was dispensed, followed by a 4 h incubation at 37 °C. DMSO (100 µL) was subsequently added to all wells to dissolve the precipitated formazan crystals. Optical density (OD) values were measured using a BMG LABTECH microplate reader (Ortenberg, Germany), and dose–response curves (six-point, normalized) were generated using GraphPad Prism 5.01 (GraphPad Software, San Diego, CA, USA). Cisplatin, a clinically used chemotherapy agent, was applied as the positive control (0.1 to 30 µM). All experiments were performed in two independent measurements with five replicates.

### 2.3. Cell Cycle Analysis Using a Flow Cytometer

To explore a potential mechanism underlying atorvastatin’s activity, cell cycle distribution was analyzed. C33A and UPCI-SCC-154 cells were seeded in 12-well plates at a 100,000–200,000 cells per well density and incubated overnight. Cells were exposed to the desired concentration of atorvastatin and incubated for 24 h and 48 h. Following this, PBS was used to wash the cells, which were subsequently trypsinized and pelleted by centrifugation at 1100 rpm for 5 min. After another wash with PBS, cells were fixed with 70% ice-cold ethanol at −20 °C for 30 min. Cells were then exposed to 300 µL of a staining mixture composed of 0.1% sodium citrate, 0.1% Triton-X, 10 µg/mL propidium iodide (PI), and 10 µg/mL RNase-A in distilled water to label their DNA content. Incubation was carried out for 30 min at ambient temperature in the dark. A flow cytometer (CytoFLEX-V0-B4-RO, Beckman Coulter, Brea, CA, USA) was used to analyze cellular DNA contents, with a cell count of no less than 20,000 ensured for each sample. The data obtained were processed using the ModFit LT 3.3.11 software (Verity Software House, Topsham, ME, USA) to evaluate the cell cycle distribution by phase-specific percentages. Untreated cells were used as controls. Hypodiploid sub-G1 phases were considered a sign of a late apoptotic event. The experiments were carried out twice, with three parallels.

### 2.4. Hoechst 33258–Propidium Iodide Fluorescent Double Staining

Apoptotic morphological changes in atorvastatin-treated cells were observed using fluorescence-based staining. UPCI-SCC-154 cells were seeded into 6-well plates at a density of 200,000 cells per well and allowed to grow overnight, followed by treatment with the test compound at multiple concentrations for 24 and 48 h durations. Then, cells were incubated for 90 min at 37 °C in a CO_2_ atmosphere with a culture medium containing 5 µg/mL of the lipophilic dye Hoechst 33258 and 3 µg/mL of the hydrophilic dye propidium iodide (PI) to visualize apoptotic and necrotic cell populations. After the replacement of the medium, a Nikon Eclipse TS100 fluorescence microscope equipped with suitable filter sets for Hoechst and PI (Nikon Instruments Europe, Amstelveen, the Netherlands) was employed to capture images, and the number of apoptotic and necrotic cells was determined by manual counting using the ImageJ software (1.53k, National Institutes of Health, Bethesda, MD, USA). The experiments were repeated twice.

### 2.5. Determination of Caspase Activity

A commercially acquired fluorometric caspase-3 detection kit (CASP3F) was employed to assess the proapoptotic effects of atorvastatin. The UPCI-SCC-154 cell line was plated in a 96-well plate at a density of 65,000 cells per well. Cells were exposed to varying concentrations of atorvastatin after an overnight incubation. Cisplatin served as the positive control for apoptosis induction. After 48 h of incubation, the culture media was discarded; lysis buffer was introduced and placed on ice for 15–20 min. The substrate of the assay kit, prepared in assay buffer, was then added to the cell lysates. Following the manufacturer’s protocol, 200 µL of each cell lysate, mixed with substrate and assay buffer, was transferred into a black-walled, clear-bottom 96-well plate. The fluorescence intensity was measured using a plate reader (CLARIOstar^Plus^, BMG LABTECH GmbH, Ortenberg, Germany) with 360 nm excitation and 460 nm emission. A control containing the same amount of reaction substrate with assay buffer accounted for background fluorescence. The level of caspase-3 activity was quantified based on the difference in fluorescence intensity between treated and control groups, and the results were expressed as fold increases in activity. The experiment was performed twice, with three replicates per condition.

### 2.6. Measurement of Mitochondrial Membrane Potential

To investigate changes in mitochondrial membrane potential, we employed the JC-10 assay. UPCI-SCC-154 cells were seeded into black 96-well plates with clear bottoms at a density of 40,000 cells per well and allowed to adhere overnight. Following a 48 h treatment period, mitochondrial staining was performed by incubating the cells with 5 μM JC-10 dye for 15 min under standard culture conditions, protected from light. After incubation, the cells were gently washed with PBS to remove excess dye. Fluorescence detection was carried out with a microplate reader. Green signals were recorded at 490 nm excitation and 525 nm emission wavelengths, whereas red fluorescence was detected at 540 nm excitation and 590 nm emission wavelengths. To assess the mitochondrial membrane potential, the ratio between red fluorescence (reflecting functioning mitochondria) and green fluorescence (indicating apoptotic activity) was quantified.

### 2.7. Antimigration Assay

Cancer cell migratory capacity in response to atorvastatin was investigated through a wound healing assay using specific silicone inserts with two separate wells (ibidi GmbH, Gräfelfing, Germany). Briefly, C33A and UPCI-SCC-154 cells were introduced into both chambers of the insert at a density of 40,000 cells per well, implanted in 12-well plates, and incubated overnight at 37 °C with 5% CO_2_ to form confluent monolayers. After carefully removing the inserts, the wells were rinsed to remove nonadherent cells and debris. Then, the cultures were incubated with the desired concentrations of atorvastatin in the medium with low FBS content (1% or 2% for C33A and UPCI-SCC-154 cells, respectively). Microscopic images were collected at baseline, 24 h, and 48 h intervals post-treatment using the Nikon Eclipse TS100 fluorescence system (Nikon Instruments Europe, Amstelveen, The Netherlands). The ImageJ software was used to determine the wound closure rate. Migration rates of treated cells were compared to those of control samples. The experiment was independently repeated twice, with three parallel wells per condition.

### 2.8. Anti-Invasion Assay

Tumor invasive potential in response to atorvastatin treatment was assessed using a three-dimensional model, the Boyden chamber assay. The assay involved using specialized Boyden chamber inserts (Corning^®^ BioCoat™ Matrigel^®^ Invasion Chamber, Bedford, MA, USA) with a polyethylene terephthalate (PET) membrane with 8 µm pores and Matrigel coating. The inserts were placed in a 24-well plate. Then, a cell suspension (100,000 and 500,000 cells per insert in serum-free EMEM for C33A and UPCI-SCC-154, respectively) containing a subinhibitory concentration of atorvastatin was added to the upper compartments. Control groups consisted of untreated cells. For C33A cells, medium enriched with 20% FBS was used, while UPCI-SCC-154 cells received a 1:1 mixture of 10% FBS-supplemented medium and NIH/3T3 fibroblast-conditioned medium; in both cases, the media were added to the lower chambers as a chemoattractant [44]. After 24 or 48 h of incubation, noninvading cells and supernatants were gently removed using a cotton swab. The remaining cells were rinsed twice with PBS, fixed in ice-cold 96% ethanol, and stained with 1% crystal violet solution for 30 min in the dark. A minimum of five images per chamber were captured using a Nikon Eclipse TS100 fluorescence microscope, and the number of invading cells was analyzed with ImageJ software. The rate of invasion was calculated through a comparison of the invaded cell number of the treated samples relative to that of the untreated control samples.

### 2.9. Statistical Analysis

Statistical analyses were performed using GraphPad Prism version 5.01 for Windows (GraphPad Software, San Diego, CA, USA). Variables are presented as mean ± SEM (or mean ± SD, where indicated). Comparisons across more than two groups were assessed by one-way ANOVA followed by Dunnett’s post hoc test to compare each treatment group with the control. Analyses of IC50 values were performed using Student’s t-tests for independent samples. All tests were two-sided and significance levels are marked by *, **, and ***, which correspond to *p* < 0.05, *p* < 0.01, and *p* < 0.001, respectively, versus the control.

## 3. Results

### 3.1. Atorvastatin Substantially Inhibited Cell Proliferation

To explore the impact of statins on the cellular proliferation of selected breast and gynecological cancer cells (MCF-7, T47-D, MDA-MB-231, HeLa, SiHa, C33A, and A2780) and OSCC cells (UPCI-SCC-154 and UPCI-SCC-131), the cells were incubated for 72 h with increasing concentrations of compounds. The inhibition of cell growth was measured using a standard colorimetric MTT assay. Breast, cervical, and OSCC cell lines were selected in accordance with their distinct HPV status or receptor profiles. Additionally, the non-cancerous MRC-5 cell line was included to assess cancer selectivity. Our results revealed that lipophilic atorvastatin exerted more pronounced growth inhibitory effects in all cell lines tested than hydrophilic rosuvastatin (Table 1). Therefore, atorvastatin was chosen for further biological investigations on HPV-negative cervical cells C33A and HPV 16-positive OSCC cells UPCI-SCC-154 based on calculated IC_50_ values and insufficient published data on the mechanism of action of atorvastatin on cancers represented by those cell lines. Concerning tumor selectivity, both tested compounds had substantially less effect on non-cancerous fibroblast MRC-5 cells than on selected cancer cell lines. Moreover, atorvastatin tumor selectivity is expressed as a ratio of calculated IC_50_ values of non-cancerous MRC-5 to tumor cell lines. Although the reference agent cisplatin elicited a generally more profound growth inhibitory action than atorvastatin, some cell lines exhibited relatively similar (C33A) or even higher sensitivity (T47-D, MDA-MB-231). Representative dose–response curves are presented in Supplementary Materials (Figures S1 and S2).

### 3.2. Atorvastatin-Induced Cell Cycle Disturbances

**Figure 1:**
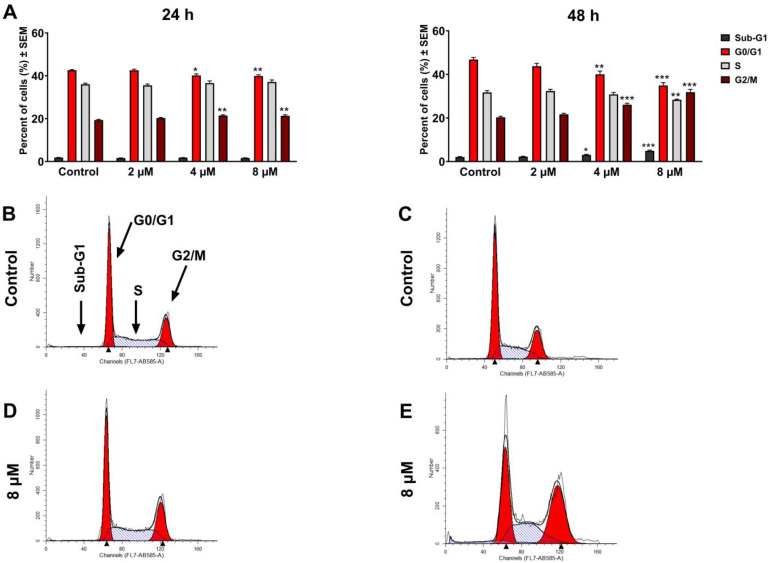
The effect of atorvastatin on the cell cycle distribution of HPV-negative cervical cancer cells C33A treated with different concentrations of the compound tested for 24 and 48 h (**A**). The results are expressed as mean values ± SEM of the data from two independent experiments performed in triplicate. *, **, and *** indicate significance at *p* < 0.05, *p* < 0.01, and *p* < 0.001, respectively, compared to the control. Representative histograms: controls after 24 h (**B**) and 48 h (**C**), 8 µM atorvastatin, incubated for 24 h (**D**) and 48 h (**E**).

Atorvastatin’s influence on cell cycle distribution was analyzed at 24 and 48 h by determining PI-stained cellular DNA. After treating C33A cells with multiple doses of atorvastatin for a 24 h period, significant cell arrest was observed in the G2/M population and a concurrent reduction in the G1 phase at 4 and 8 μM. Extended exposure for 48 h resulted in a dose-dependent elevation in the G2/M phase, accompanied by suppression of the G1 phase, with a modest suppression in the S phase progression. In addition, a marked elevation in the sub-G1 hypodiploid cell population was observed. Notably, the lowest tested 2 μM concentration of atorvastatin did not cause substantial features of cell cycle disturbance in cervical C33A cells at the two tested time intervals (Figure 1 and Figure S3).

**Figure 2:**
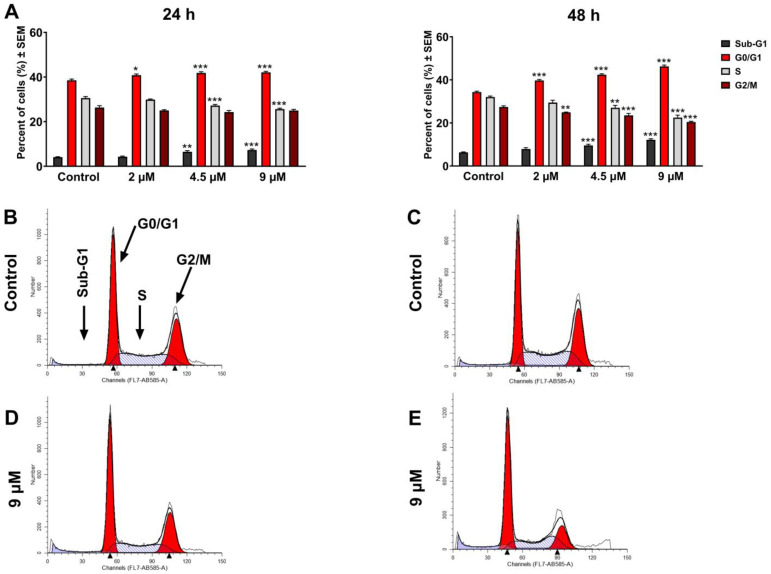
The effect of atorvastatin on the cell cycle distribution of HPV-positive OSCC cells UPCI-SCC-154 treated with different concentrations of the compound tested for 24 and 48 h (**A**). The results are expressed as mean values ± SEM of the data from two independent experiments performed in triplicate. *, **, and *** indicate significance at *p* < 0.05, *p* < 0.01, and *p* < 0.001, respectively, compared to the control. Representative histograms: controls after 24 h (**B**) and 48 h (**C**), 9 µM atorvastatin, incubated for 24 h (**D**) and 48 h (**E**).

Meanwhile, our results in OSCC cells (UPCI-SCC-154) indicated a remarkable concentration-based accumulation of the G1 phase associated with S phase depletion and a notable accumulation of cells in the sub-G1 phase following 24 h incubation. Nonetheless, a longer 48 h incubation led to more pronounced effects on cell cycle distribution, accompanied by a reduction in the G2/M phase (Figure 2 and Figure S4).

### 3.3. Atorvastatin-Induced Cellular Apoptosis Visualized by Fluorescent Double Staining

**Figure 3:**
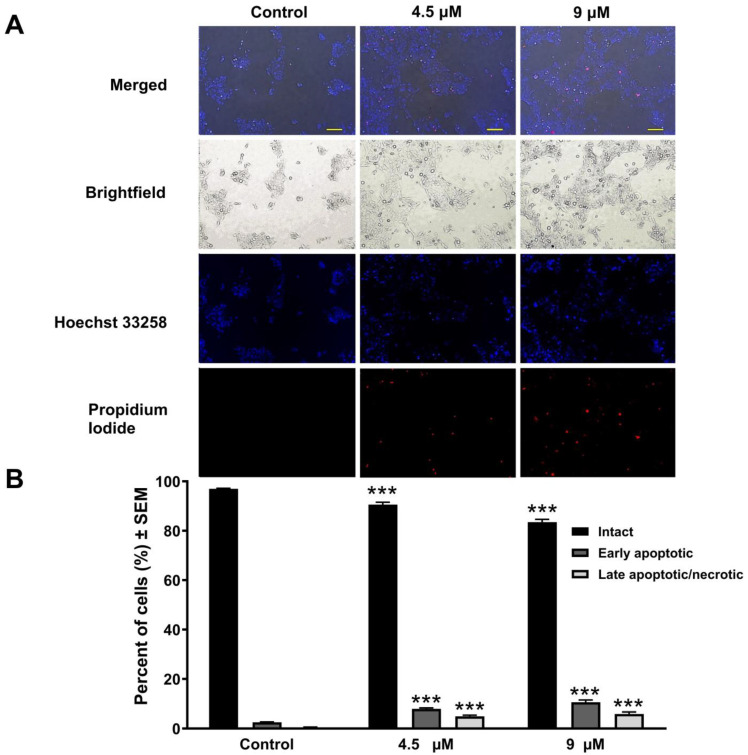
Morphological alterations of UPCI-SCC-154 cells after 24 h of treatment with atorvastatin were observed using Hoechst/PI double staining. Cell nuclei were stained with Hoechst 33258 (blue fluorescence) and propidium iodide (red fluorescence); image pairs were taken from the same field (**A**). The bar in the images indicates 100 µm. The graph indicates percentages of intact, early apoptotic, and late apoptotic/necrotic cell populations (**B**). The results are presented as mean values ± SEM of the data from two independent experiments performed in duplicates. *** indicating significance at *p* < 0.001 compared to control.

**Figure 4:**
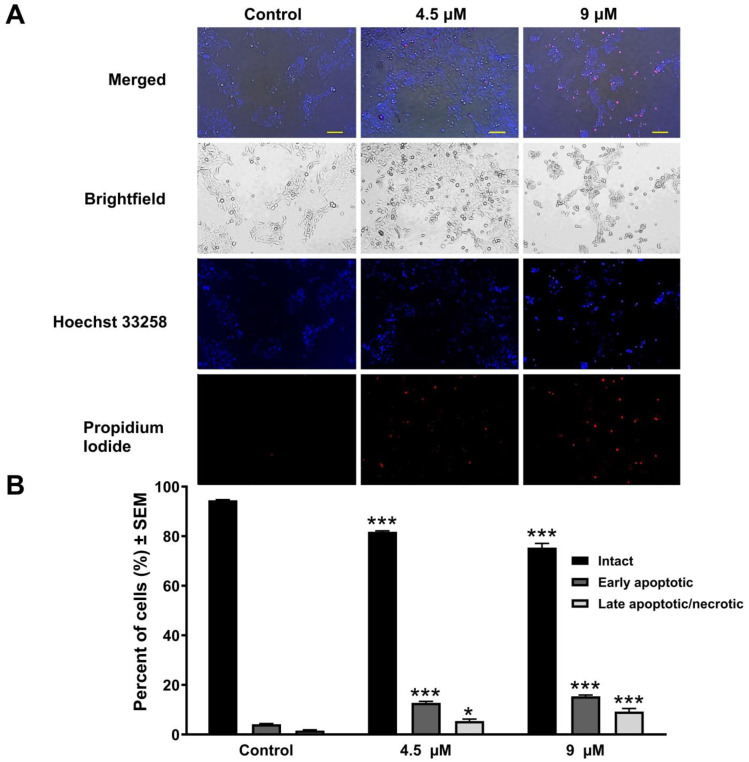
Morphological alterations of UPCI-SCC-154 cells after 48 h of treatment with atorvastatin were observed using Hoechst/PI double staining. Cell nuclei were stained with Hoechst 33258 (blue fluorescence) and propidium iodide (red fluorescence); image pairs were taken from the same field (**A**). The bar in the images indicates 100 µm. The graph indicates percentages of intact, early apoptotic, and late apoptotic/necrotic cell populations (**B**). The results are presented as mean values ± SEM of the data from two independent experiments performed in duplicates. *, and *** indicating significance at *p* < 0.05 and *p* < 0.001, respectively, compared to control.

The exposure of UPCI-SCC-154 cell lines to different atorvastatin concentrations for 24 and 48 h resulted in notable alterations in morphological features and the integrity of the cellular membrane. Fluorescent nuclei staining with Hoechst and PI dyes showed a reduction in the number of intact cells in relation to the applied concentration. Additionally, a marked increase in nuclei displaying intense blue fluorescence, suggestive of DNA condensation and membrane blebbing, indicates early apoptosis. A noticeable number of cells in addition emitted red fluorescence, especially after a longer incubation period, suggesting the presence of secondary necrosis, which points to potential damage to the cell membranes (Figure 3 and Figure 4).

### 3.4. Atorvastatin Promotes Apoptosis by Activating Caspase-3

**Figure 5:**
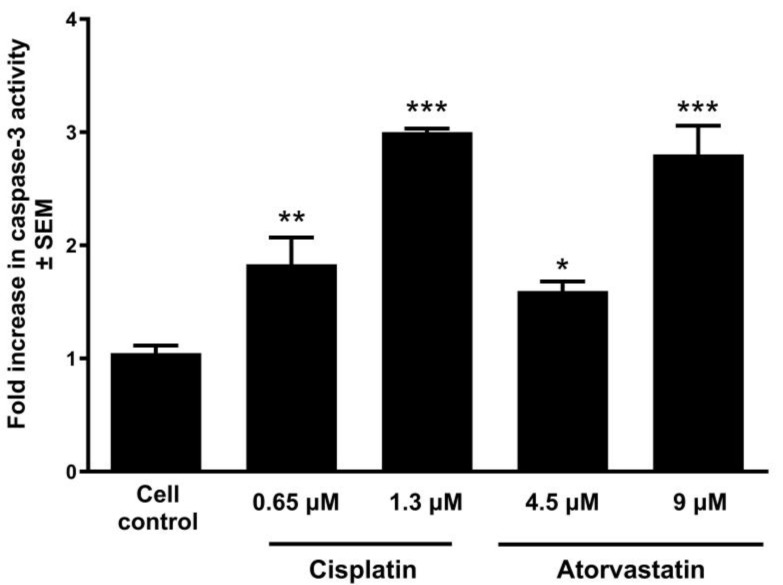
Caspase-3 activity measurement of UPCI-SCC-154 cells after 48 h exposure to atorvastatin. The results are expressed as mean values ± SEM of the data from two independent experiments performed in triplicate. *, **, and *** indicate significance at *p* < 0.05, *p* < 0.01, and *p* < 0.001, respectively, compared to the control samples.

After evaluating the morphological alterations and cell cycle progression, caspase-3 enzymatic activity, a key effector in apoptosis, was measured following 48 h exposure to varying doses of atorvastatin. This study used cisplatin as a positive reference, and the concentrations used were determined in accordance with the previously calculated IC_50_ values. The findings revealed that atorvastatin increased the enzyme activity in a manner dependent on its concentration in a relatively similar manner to the positive control (Figure 5). Importantly, the observed effects were rescued by co-treatment with a caspase-3 inhibitor, supporting the involvement of caspase-3-mediated apoptosis (Figure S5).

### 3.5. Atorvastatin-Induced Mitochondrial Membrane Damage

**Figure 6:**
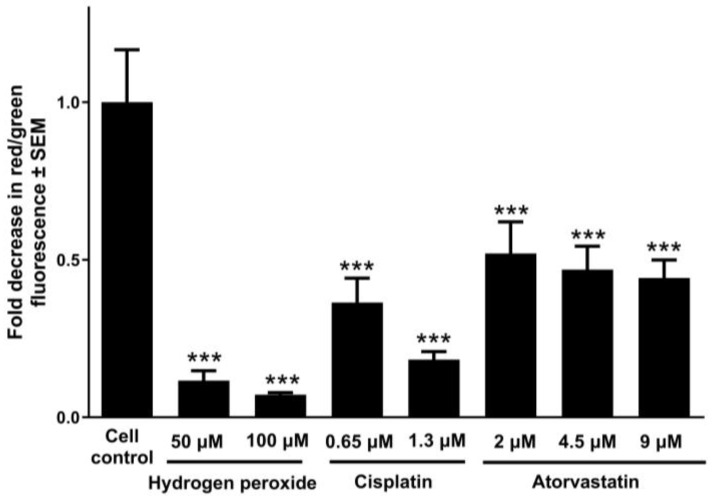
The mitochondrial membrane potential of UPCI-SCC-154 cells after 48 h exposure to atorvastatin using JC-10 staining. The results are expressed as mean values ± SEM of the data from three independent experiments, with a minimum of three replicates. *** indicates significance at *p* < 0.001 compared to the control samples.

To further elucidate the role of mitochondrial impairment in atorvastatin-induced apoptosis, we assessed mitochondrial membrane potential in UPCI-SCC-154 cells using JC-10 dye. Cells were exposed to increasing concentrations of atorvastatin, while hydrogen peroxide and cisplatin were positive controls for mitochondrial disruption. The analysis revealed a dose-dependent decline in mitochondrial membrane potential following atorvastatin treatment, as evidenced by a progressive reduction in the red-to-green fluorescence ratio, indicative of enhanced mitochondrial depolarization (Figure 6).

### 3.6. Atorvastatin-Mediated Suppression of Cell Migration

**Figure 7:**
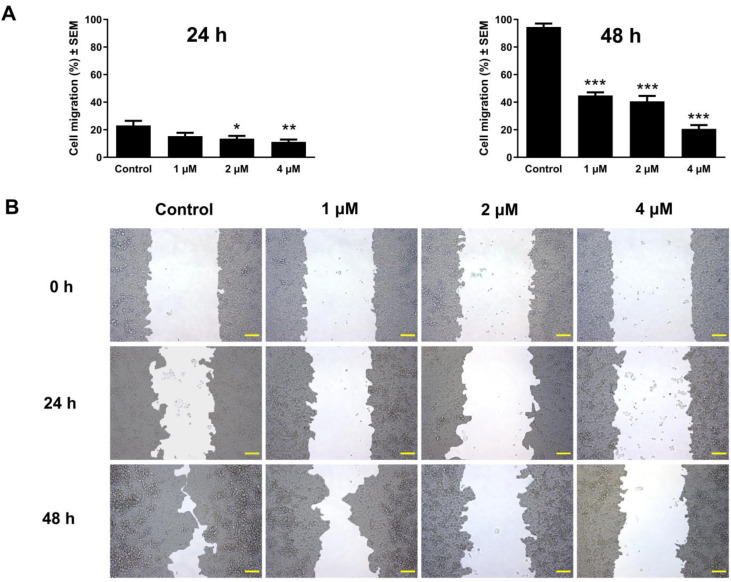
The effect of atorvastatin on the migration of cervical cancer cells C33A. Graphs indicate the percentage of cell migration at 24 and 48 h post-treatment of C33A cells with 1, 2, and 4 μM of atorvastatin relative to the control (**A**). Representative images of wound closure at 0, 24, and 48 h post-treatment (**B**). The bar in the photos indicates 100 µm. The results are presented as mean values ± SEM of the data from two independent measurements, with triplicate measurements. *, **, and *** indicating significance at *p* < 0.05, *p* < 0.01, and *p* < 0.001, respectively, compared to the control.

**Figure 8:**
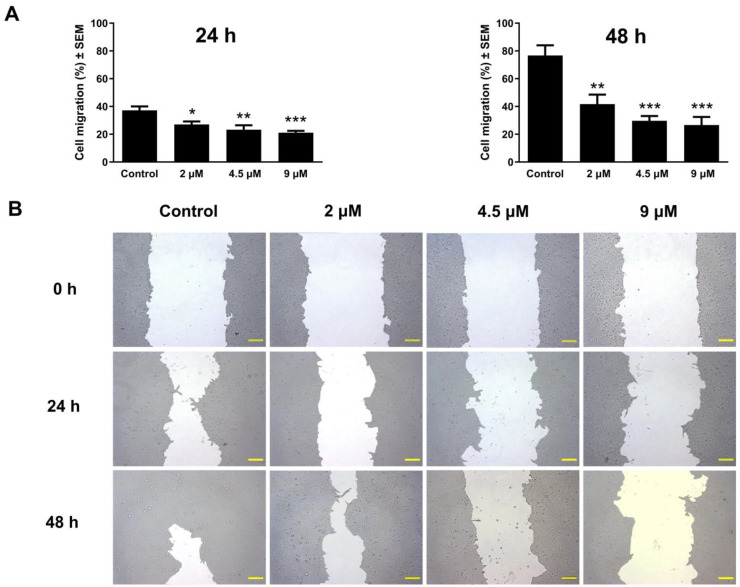
The effect of atorvastatin on the migration of OSCC cells. The graphs indicate the percentage of cell migration at 24 h and 48 h post-treatment of UPCI-SCC-154 cells with 2, 4.5, and 9 μM of atorvastatin relative to the control (**A**). Representative images of wound closure at 0, 24, and 48 h post-treatment (**B**). The bar in the photos indicates 100 µm. The results are presented as mean values ± SEM of the data from two independent measurements made in triplicate. *, **, and *** indicating significance at *p* < 0.05, *p* < 0.01, and *p* < 0.001, respectively, compared to the control.

To assess the antimigratory effect of atorvastatin, the area devoid of cellular coverages was evaluated at three different time intervals (0, 24, and 48 h) after treatment in a concentration range from 1 to 9 μM under low-serum culture conditions. The closure of these cell-free areas was monitored by image analysis. The results showed a decrease in wound closure in a manner dependent on both exposure time and concentration at 24 and 48 h post-incubation relative to the untreated control. These findings were consistent in the tested cell lines C33A and UPCI-SCC-154, as shown in Figure 7 and Figure 8.

### 3.7. Atorvastatin Demonstrated Substantial Anti-Invasive Activity

**Figure 9:**
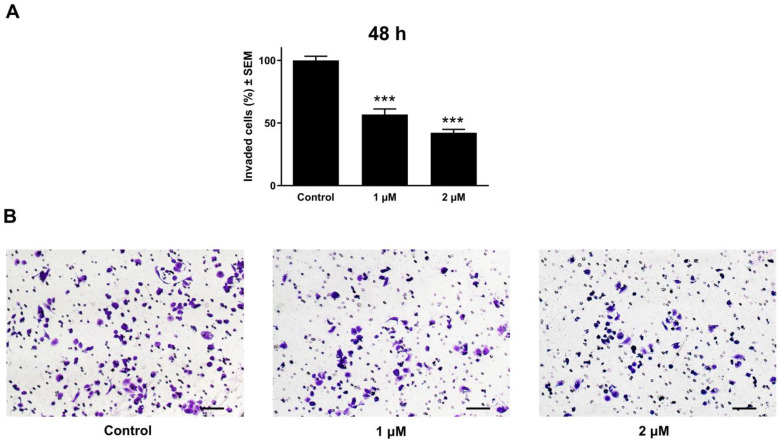
The effect of atorvastatin on the invasiveness of cervical cancer cells (C33A). The percentage of invading cells after atorvastatin treatment at 1 and 2 μM (**A**). The anti-invasive potential of the test compound is illustrated by representative images taken 48 h post-treatment (**B**). The bar in the photos indicates 100 µm. The results are expressed as mean values ± SEM of the data from two independent measurements with duplicates. *** indicates *p* < 0.001 compared to the control.

**Figure 10:**
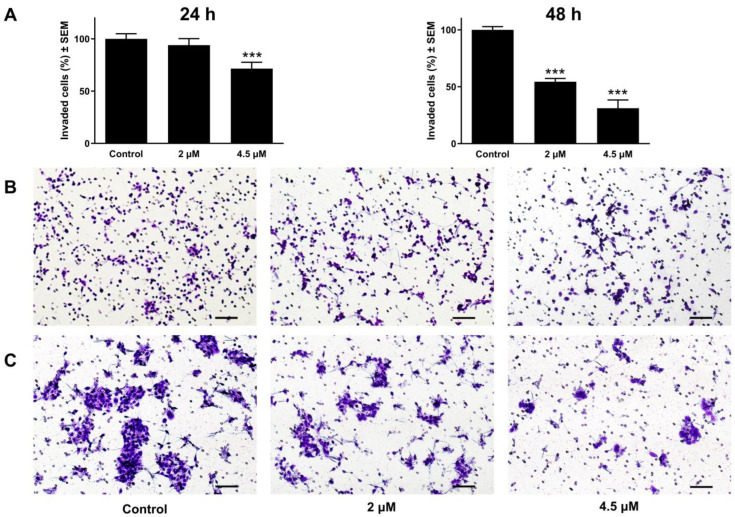
The effect of atorvastatin on the invasiveness of OSCC cells (UPCI-SCC-154). The percentage of invading cells after atorvastatin treatment at 2 and 4.5 μM (**A**). The anti-invasive potential of the test compound is illustrated by representative images taken 24 h (**B**) and 48 h (**C**) post-treatment. The bar in the photos indicates 100 µm. The results are expressed as mean values ± SEM of the data from two independent measurements with duplicates. *** indicates *p* < 0.001 compared to the control.

Cancer cell invasion, in addition to migration, is an integral part of cancer metastasis. Matrigel-coated transwell inserts were used to evaluate cancer cell invasive capacity. Images were captured for each insert, allowing for the number of penetrated cells to be counted. The obtained cell count was used to calculate the proportion of cancer cells that invaded the membrane. Relative to the untreated controls, the C33A cervical cell line treated with atorvastatin showed a marked and concentration-influenced reduction in cancer cell invasion at tested concentrations (1 and 2 μM), 48 h post-incubation (Figure 9). In contrast, the highly invasive oral UPCI-SCC-154 cells were tested at 24 and 48 h post-incubation. A modest action was observed in a reduced incubation time of 24 h. However, following extended incubation for 48 h, a noticeable reduction in the invasive cell density was observed (Figure 10).

## 4. Discussion

Cancer continues to be a critical worldwide health issue, with high mortality rates and a significant impact on global healthcare systems, emphasizing the urgent need for more advanced strategies with safer and more effective agents. Statins have been used for decades to treat dyslipidemia and decrease cardiovascular complications and mortality. They work by inhibiting HMG-CoA reductase in the hepatic mevalonate pathway, a crucial enzyme for cholesterol synthesis. This pathway also influences various cellular processes, suggesting that its inhibition may contribute to potential anticancer effects. Additionally, statins might demonstrate antitumor activities through mevalonate-independent mechanisms [14,40,45,46].

Although statins have shown anticancer properties in various types of cancer [47,48,49,50,51], there are still limited data regarding their impact on cervical cancer and OSCC cells. Further research is necessary to reveal statins with greater potency and improved safety profiles for future clinical trials.

The current study examined the antiproliferative properties of two commonly prescribed statins with different physicochemical characteristics, atorvastatin and rosuvastatin [52]. The growth-inhibitory assay was performed on a panel of adherent human cancer cells of varying origins, receptors, and HPV statuses. Our data revealed that atorvastatin was more potent than rosuvastatin in all treated cells, as significant growth-inhibitory effects were achieved at lower concentrations (lowest calculated IC_50_ value: 2.57 µM). This finding is correlated with other studies that have investigated the growth-inhibitory effects of several statins, including simvastatin, lovastatin, pravastatin, atorvastatin, and rosuvastatin [50,53,54]. Variations in the chemical structure and cell entry mechanisms (through passive membrane diffusion or specific transporters) could explain the differences in the anticancer effects observed between different statins [55]. Interestingly, the antiproliferative results of atorvastatin for some cancer cell lines were relatively comparable to those of the standard therapy, cisplatin. Since statins are generally well-tolerated drugs, their action on cancer cell viability appears more relevant than expected based solely on calculated IC_50_ values. Under the same experimental conditions, tumor selectivity was determined by testing the compounds on non-cancerous fibroblast cells, MRC-5. Notably, the concentrations applied in our in vitro investigations (0.3–100 µM) are higher than those achieved with standard statin therapy for the treatment of dyslipidemia (10–100 nM) [56]. Although regularly utilized statin doses would not be expected to produce the observed effects, a study showed that high-dose lovastatin therapy (45 mg/kg/day) can reach plasma levels of approximately 4 µM [57], which falls within the dose range used in our experiments; noteworthy, the study highlighted that the utilization of ubiquinone was associated with the prevention and reversal of lovastatin-induced myopathy. Furthermore, earlier clinical research has not associated the anticancer properties of statins with the doses needed to inhibit cholesterol production. Therefore, statins might produce anticancer activity in vivo at even lower concentrations [58]. Nonetheless, alternative delivery strategies (e.g., intratumoral injection or nanoparticles), which have been explored in related contexts [59], may achieve higher local concentrations. Thus, our findings should be considered hypothesis-generating, particularly for use in combination strategies. Prior evidence of radiosensitization and chemosensitization supports the potential for rational combinations that could enhance efficacy [34,60,61]. Considering the tumor selectivity and specificity findings, HPV-negative cervical cancer cells C33A and HPV-positive OSCC cells UPCI-SCC-154 have been selected for further investigations. By investigating the anticancer properties of atorvastatin in these distinct cell lines, we can gain a deeper understanding of its mechanism of action among cells with different HPV statuses, supporting a broader perspective on personalized medicine in cancer treatment.

Statins influence the expression and activity of cyclins, cyclin-dependent kinases (CDKs), and CDK inhibitors [62], resulting in a blockade of cell cycle progression at the G1/S and G2/M phases [63,64]. This cell cycle blockade may be related to stabilizing CDK inhibitors p21 and p27 [65]. Furthermore, the cell cycle is disrupted when Ras farnesylation is inhibited [66] or the levels of geranylgeranylated proteins are reduced, as these proteins are vital for the initiation of the S-phase [67]. In the present study, to explore the underlying mechanism by which atorvastatin inhibits the proliferation of cervical cancer cells and OSCC cells, we determined its impact on the cell cycle via flow cytometric analysis. The findings revealed that it caused disturbances in the cell cycle progression in the HPV-negative cervical cancer cell line C33A, marked by an elevated proportion of the cell population in the G2/M phase and a suppression in the cell population within the G0/G1 phase in a concentration- and time-influenced manner. Meanwhile, 24 h of HPV-positive UPCI-SCC-154 cells exposure to atorvastatin led to cell cycle arrest at the G0/G1 phase alongside depletion of the S phase. An extended 48 h incubation demonstrated more pronounced cell cycle disturbance features accompanied by G2/M phase suppression. Noteworthy, there was a significant elevation in the hypodiploid cells in both tested cell lines, indicating that atorvastatin may promote apoptotic cell death [68]. However, the effects are more substantial in the OSCC cell line since the concentrations tested did not exceed the inhibitory concentration. Our findings might reflect an influence of mevalonate pathway. Although we did not directly investigate this mechanism, future studies could employ reversal experiments with mevalonate, farnesyl pyrophosphate, or geranylgeranyl pyrophosphate to determine whether the observed effects are mediated through impaired protein prenylation.

Statins activate the intrinsic apoptosis pathway and reduce Bcl-2 protein expression [69], raise the levels of Bax and BIM protein [70], and trigger the activation of numerous caspases [19,50,71]. Under favorable conditions, disrupting the balance between pro- and anti-apoptotic proteins results in statin-mediated apoptotic cell death. Additionally, the extrinsic apoptosis cascade could be an essential determinant in the antitumor effects of statins through the upregulation of Fas [54]. In our study, Hoechst/PI fluorescence double staining confirmed the apoptosis-inducing effect of atorvastatin detected by flow cytometry analysis. The OSCC cell line has been selected for further investigation based on the more pronounced apoptotic sub-G1 population induced by atorvastatin. Nuclear condensation and membrane blebbing, signs of early apoptotic cell death [72], were observed with fluorescent Hoechst staining in UPCI-SCC-154 cells 24 and 48 h post-incubation with varying atorvastatin concentrations. Additionally, atorvastatin resulted in late cellular apoptosis or secondary necrosis in treated cells, characterized by compromised membrane integrity as visualized by PI staining, evidenced by the red fluorescence observed in the cells. Importantly, atorvastatin exposure was also associated with a significant loss of mitochondrial membrane potential, suggesting that mitochondrial dysfunction plays a key role in atorvastatin-induced apoptosis. The disruption of mitochondrial integrity is a hallmark of the intrinsic apoptotic pathway, which in turn activates downstream effector caspases. Consistently, a significant increase in caspase-3 activity was detected in UPCI-SCC-154 cells following 48 h exposure to atorvastatin. These findings strongly suggest that atorvastatin induces apoptosis in OSCC cells via mitochondrial-initiated caspase activation, supporting its potential as an effective proapoptotic agent.

During tumor progression, particularly in the development of metastases, cell migration is vital in facilitating the tumor spread and the emergence of distant metastases. To reduce mortality and morbidity associated with cancer, there is a persistent need for research on drug candidates that can disrupt metastasis formation [73,74]. In our in vitro study, atorvastatin exhibited a significant capacity to hinder the migration of C33A and OSCC cell lines, with effects modulated by both exposure duration and concentration. Remarkably, even at subinhibitory concentrations, a considerable inhibitory effect was observed after 24 and 48 h of incubation. Previous studies reported that statins disturb cancer migration and adhesion and subsequently reduce cancer cell metastases [75,76]. Evidence indicates that the antimetastatic effect of statins is mediated through their interference with the geranylgeranylation and farnesylation processes of small GTPases [77,78]. Furthermore, a study found that atorvastatin increases reactive oxygen species (ROS) in the OSCC cells, thereby inhibiting cell migration [79]. Studies have shown that statins inhibit EMT, a mechanism that potentially involves the metastasis-suppressing effects of statins [80,81].

In addition to cell migration, the invasion and infiltration of tumor cells into adjacent tissues are critical to metastasis. To complement our findings from the wound healing assay focused on cellular motility, we included a Boyden chamber invasion assay, which is expected to replicate the tumor-associated extracellular microenvironment at the primary site. Our results showed that atorvastatin greatly reduced cell invasive potential proportionally with compound concentration 48 h post-incubation in the two tested cell lines, with modest anti-invasive effects 24 h in OSCC cells. Interestingly, the inhibition of migration and invasion at sub-IC_50_ concentrations, which are close to achievable plasma levels, may be pharmacologically relevant even in the absence of systemic antiproliferative effects. The inhibition of invasion by statins in the aggressive MDA-MB-231 triple-negative breast cancer cells has been attributed to the suppression of the RhoA/ROCK/NF-kB signaling pathway [65]. In melanoma cells, it was suggested that atorvastatin could block cell invasion by altering endogenous Rho signaling [77], while in glioma cells, atorvastatin reduces cell invasiveness by suppressing the microglial membrane type 1 metalloproteinase (MT1-MMP) expression [82].

## 5. Conclusions

There has been a recent increase in advances towards personalized medicine approaches in cancer therapy. Our experimental findings demonstrate the potential antiproliferative and antimetastatic properties of atorvastatin on HPV-negative cervical cancer cells C33A and HPV-positive OSCC cells UPCI-SCC-154. While the results are encouraging, translation into clinical use will require comprehensive in vivo studies, a detailed pharmacokinetic profile, and an evaluation of its clinical relevance as a repositioned drug.

## Figures and Tables

**Table 1:** Growth-inhibitory effects of atorvastatin and rosuvastatin on the cell lines tested.

Cell Lines	Atorvastatin	Rosuvastatin	Cisplatin	**Atorvastatin** Tumor Selectivity
	IC_50_ (μM) ± SD			
MCF-7	61.01 ± 3.71 **	100<	8.19 ± 0.20	0.80
T47-D	8.32 ± 1.52 *	100<	18.36 ± 1.24	5.85
MDA-MB-231	2.57 ± 0.30 ***	18.22 ± 1.12	19.12 ± 0.02	18.96
HeLa	20.27 ± 0.73 **	64.91 ± 1.80	12.43 ± 0.20	2.40
SiHa	12.02 ± 2.42	38.39 ± 0.30	4.80 ± 0.72	4.05
C33A	4.61 ± 0.15	31.63 ± 0.78	4.70 ± 1.64	10.54
A2780	4.02 ± 0.78 *	96.13 ± 0.72	1.34 ± 0.05	12.11
UPCI-SCC-154	9.21 ± 1.68 *	94.09 ± 0.86	1.29 ± 0.001	5.28
UPCI-SCC-131	34.74 ± 1.63 **	94.16 ± 1.80	1.37 ± 0.21	1.40
MRC-5	48.64 ± 3.24 **	100<	4.74 ± 0.32	

Between-group comparisons were performed using two-sided Student’s t-tests for independent samples. *, **, and *** indicate significance at *p* < 0.05, *p* < 0.01, and *p* < 0.001, respectively, compared to cisplatin.

## Data Availability

The datasets of the current study are available from the corresponding authors on reasonable request.

## Supplementary Materials

The following supporting information can be downloaded at https://www.mdpi.com/article/10.3390/pharmaceutics17101253/s1, Figure S1: Dose–response curves of antiproliferative activity for atorvastatin and rosuvastatin. Figure S2: Dose–response curves of antiproliferative activity for cisplatin. Figure S3: Representative dot plots of atorvastatin-induced cell cycle arrest in the C33A cells. Figure S4: Representative dot plots of atorvastatin-induced cell cycle arrest in the UPCI-SCC-154 cells. Figure S5: Caspase-3 activity measurement of UPCI-SCC-154 cells after 48 h exposure to atorvastatin.

## Abbreviations

The following abbreviations are used in this manuscript:

HMG-CoA	3-Hydroxy-3-methylglutaryl coenzyme A
EMT	Endothelial–mesenchymal transition
VEGF	Vascular endothelial growth factor
OSCC	Oral squamous cell carcinoma
DMSO	Dimethylsulfoxide
ECACC	European Collection of Authenticated Cell Cultures
ATCC	American Tissue Culture Collection
EMEM	Eagle’s Minimal Essential Medium
FBS	Fetal bovine serum
NEAA	Non-essential amino acid
DMEM	Dulbecco’s Modified Eagle Medium
PBS	Phosphate-buffered saline
PI	Propidium iodide
ROS	Reactive oxygen species
MT1-MMP	Microglial membrane type 1 metalloproteinase
