# Supplementary material for: In Vitro Investigation of the Antiproliferative and Antimetastatic Effects of Atorvastatin: A Focus on Cervical and Head and Neck Cancers
Source: Pharmaceutics. 2025 Sep 24;17(10):1253. doi: 10.3390/pharmaceutics17101253 (PMC12567309; doi:10.3390/pharmaceutics17101253)
Supplement: Supplementary file 1 [file pharmaceutics-17-01253-s001.zip › pharmaceutics-3867249-supplementary.pdf]

Supplementary Materials

In Vitro Investigation of the Antiproliferative and Antimetastatic Effects of Atorvastatin: A Focus on Cervical and Head and Neck Cancers

Hiba F. Muddather[1,2], Noémi Bózsity[1], György T. Balogh[1,3], Zsuzsanna Schelz[1,*] and István Zupkó[1,*]

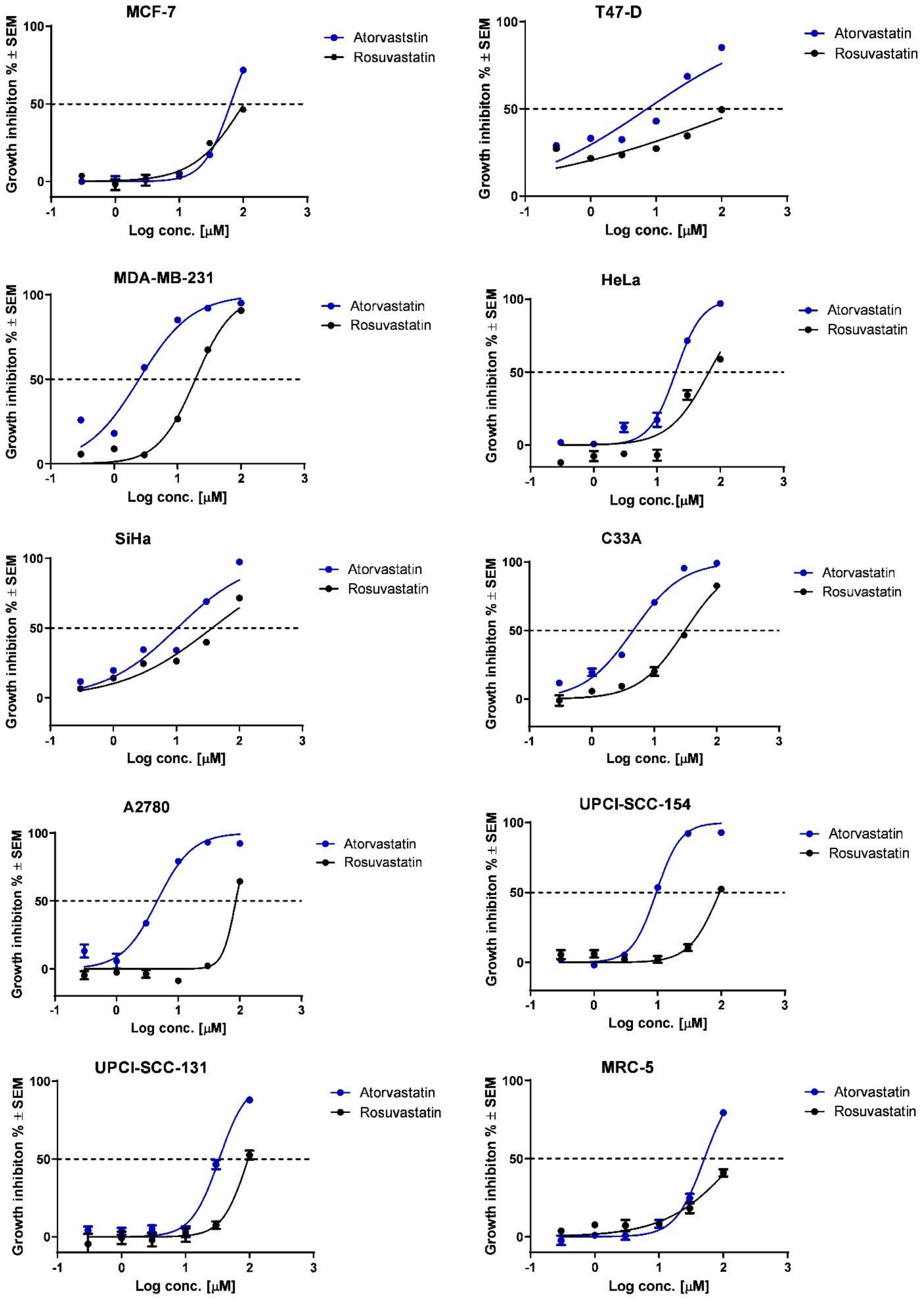

Figure S1. Dose–response curves of antiproliferative activity for atorvastatin and rosuvastatin

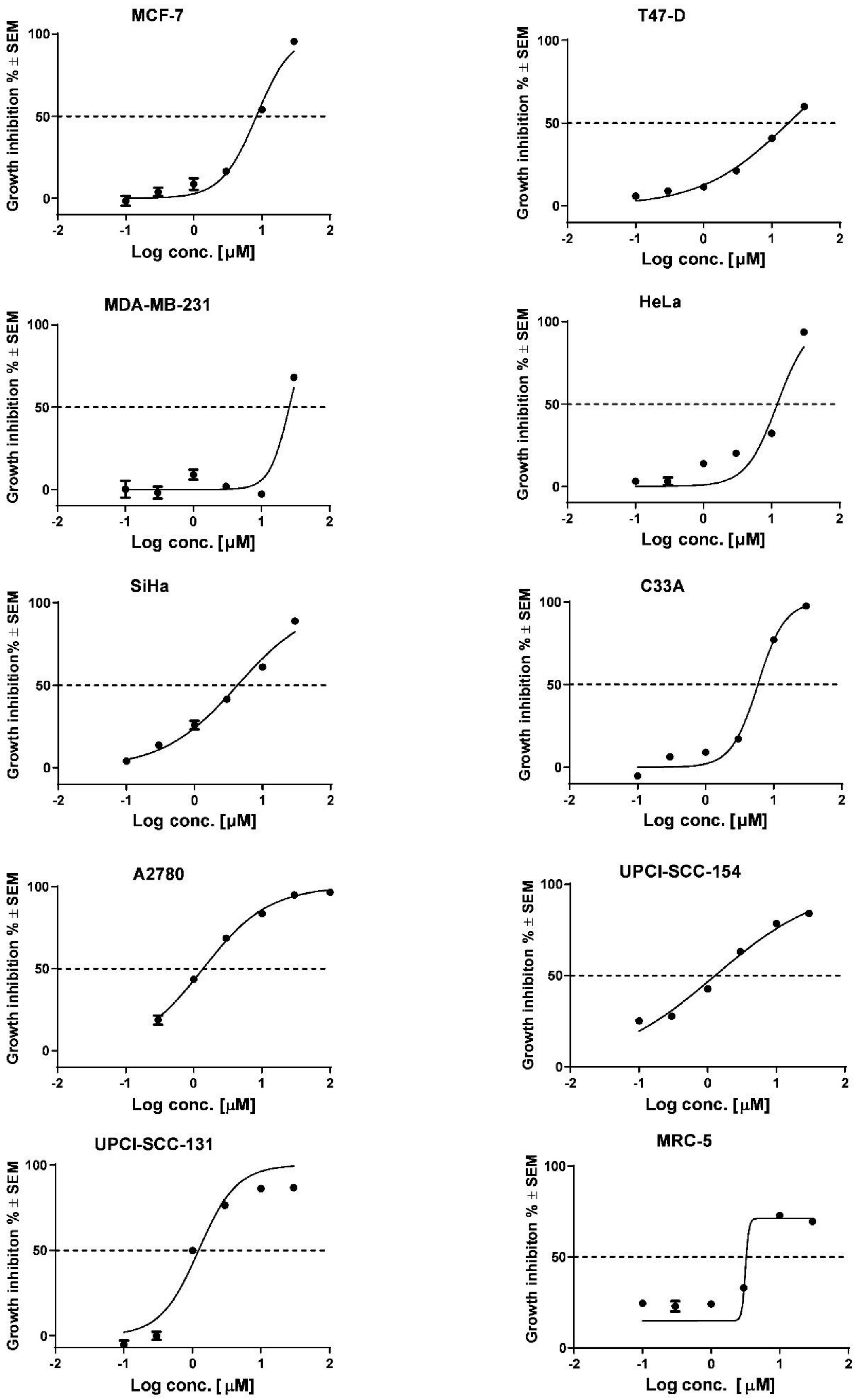

Figure S2. Dose–response curves of antiproliferative activity for cisplatin

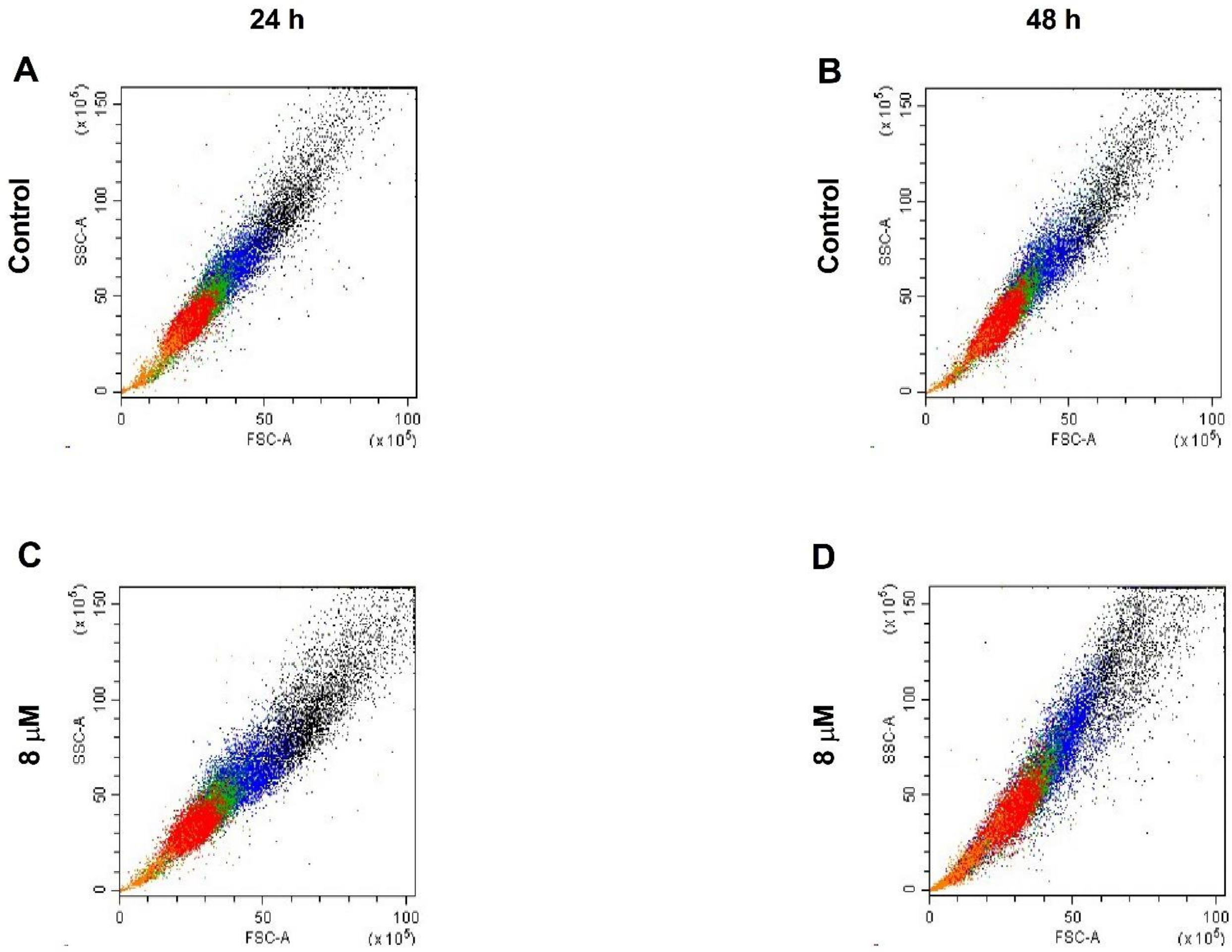

Figure S3. Atorvastatin induces cell cycle arrest in the C33A cells. Representative dot plots: Controls **(A, B)** 24 h and 48 h, respectively, and treated cells **(C, D)** 24 h and 48 h post-treatment, respectively. Colors indicate cell cycle phases: yellow (Sub-G1), red (G1), green (S), and blue (G2/M).

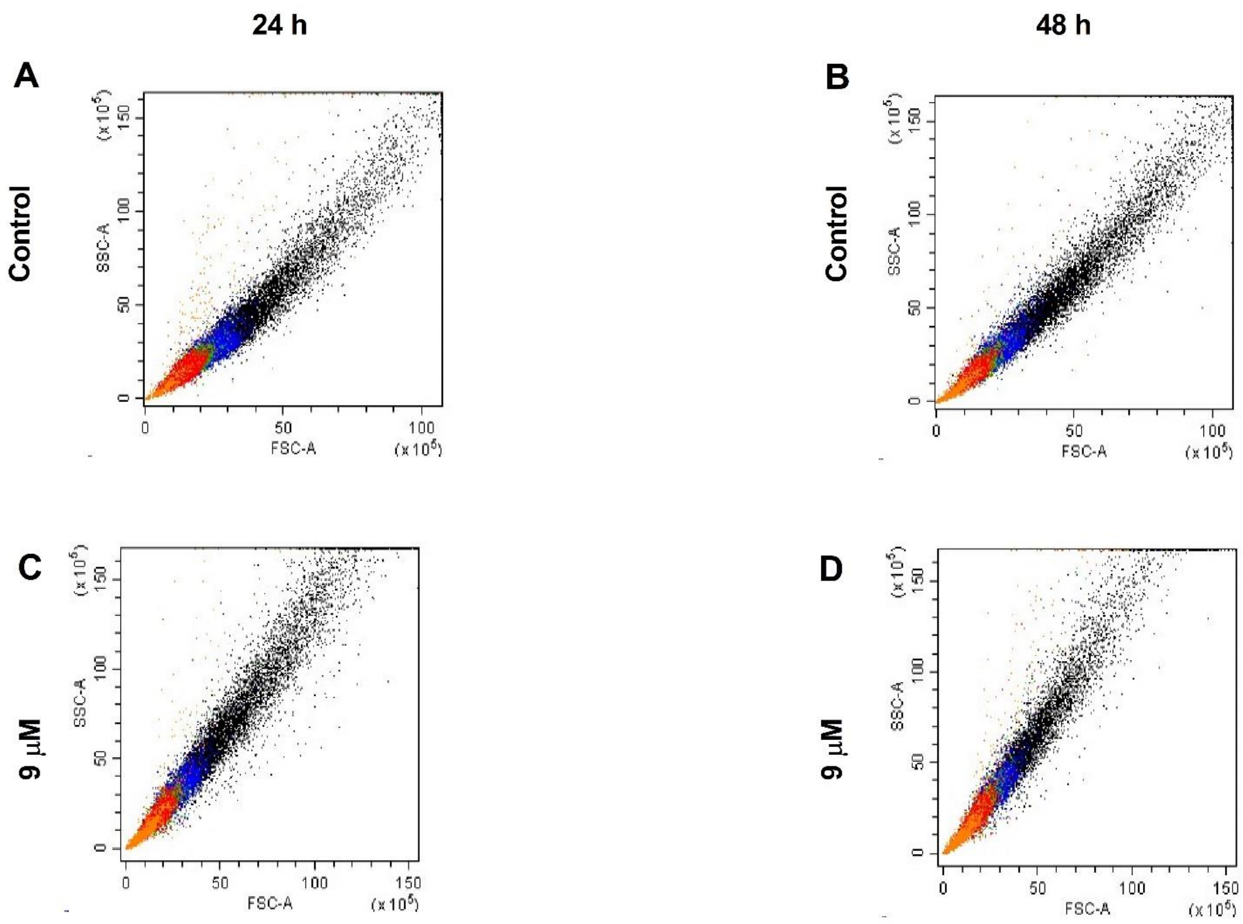

Figure S4. Atorvastatin induces cell cycle arrest in the UPCI-SCC-154 cells. Representative dot plots: Controls **(A, B)** 24 h and 48 h, respectively, and treated cells **(C, D)** 24 h and 48 h post-treatment, respectively. Colors indicate cell cycle phases: yellow (Sub-G1), red (G1), green (S), and blue (G2/M).

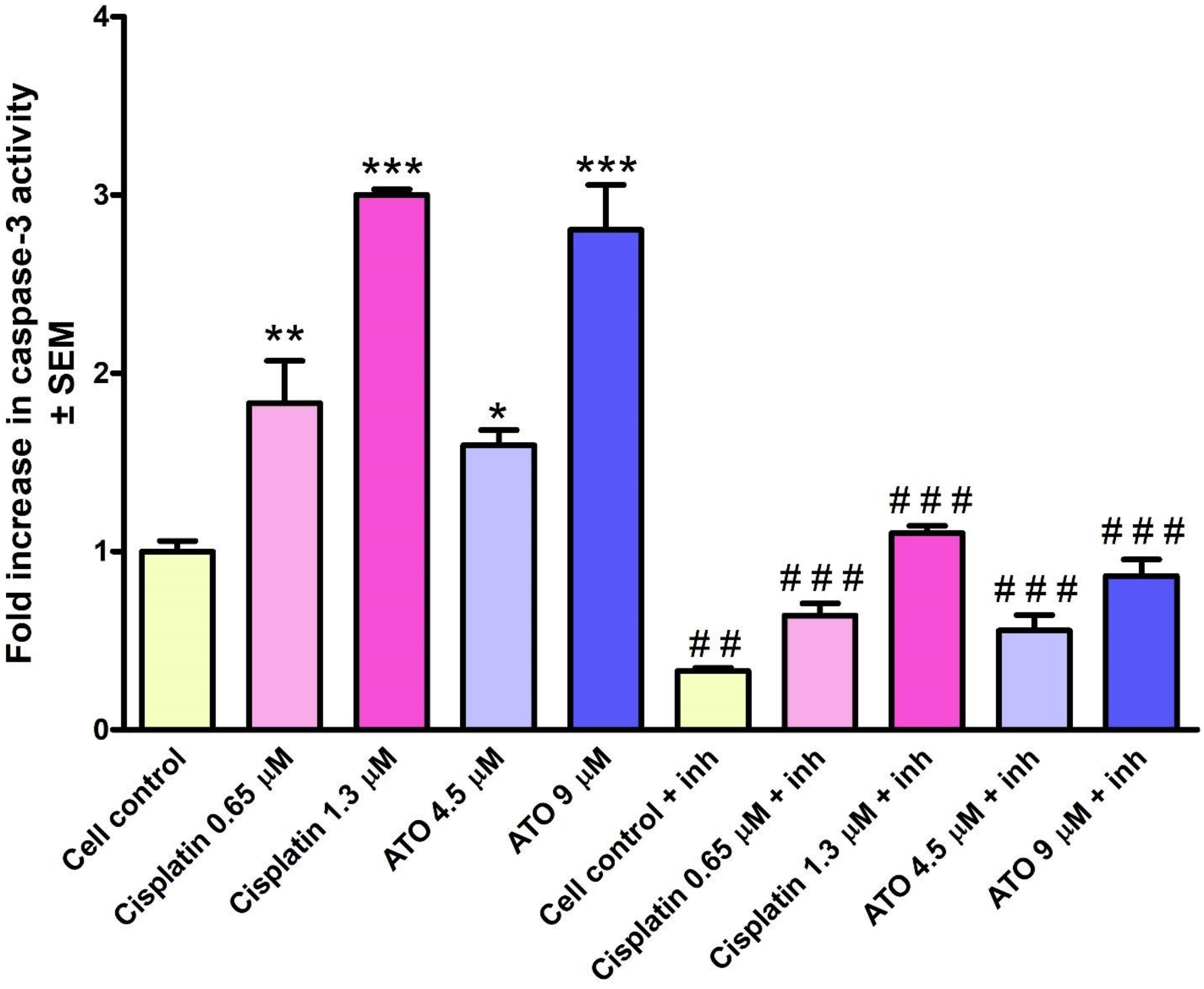

Figure S5. Caspase-3 activity measurement of UPCI-SCC-154 cells after 48 h exposure to atorvastatin. Results are expressed as mean values ± SEM of the data from two independent experiments performed in triplicate. *, **, and *** indicate significance at $p < 0.05$, $p < 0.01$, and $p < 0.001$, respectively, compared to control samples. ##, and ### indicate significance at $p < 0.01$, and $p < 0.001$, respectively, compared to the corresponding condition without caspase-3 activity inhibition. ATO, atorvastatin; inh, inhibitor.

Formulas and Calculations:

$$\text{Inhibition \%} = \frac{\text{Absorbance}_{\text{Sample}} - \text{Absorbance}_{\text{Blank}}}{\text{Absorbance} - \text{Absorbance}_{\text{Blank}}} \times 100$$

$$\text{Wound closure} = 100 - \frac{\text{Wound surface}_{\text{after 24/48 h}}}{\text{Wound surface}_{\text{at 0 h}}} \times 100$$
